# Is POC-CCA a truly reliable test for schistosomiasis diagnosis in low endemic areas? The trace results controversy

**DOI:** 10.1371/journal.pntd.0006813

**Published:** 2018-11-08

**Authors:** José M. Peralta, Marta G. Cavalcanti

**Affiliations:** 1 Departmento de Imunologia, Instituto de Microbiologia Paulo de Góes, Universidade Federal do Rio de Janeiro, Rio de Janeiro, Brazil; 2 Serviço de Doenças Infecciosas e Parasitárias, Hospital Universitário Clementino Fraga Filho, Universidade Federal do Rio de Janeiro, Rio de Janeiro, Brazil; George Washington University, UNITED STATES

## Introduction

In the last decade, development of point-of-care platforms (POCs) for diagnosis of infectious diseases or rapid tests (RTs) has increased in order to attend to the need for reliable diagnostic tests for surveillance of endemic areas. Also, preventive chemotherapy in areas without sophisticated resources has become more affordable according to some field studies [1,2]. The advantages of application of RTs such as POC-*Schistosoma* circulating cathodic antigen (CCA) are undeniable in community settings. But some evidence also pointed to other uses, like in institutional settings, by investigating active infections in susceptible populations like travelers, immigrants, and/or refugees [3,4]. Nonetheless, a great deal of discussion still remains toward the exclusive use of the RTs and/or POCs as tools for monitoring schistosomiasis in controlled areas or areas undergoing elimination. Despite the obvious advantages, the growing application of RTs has also shown some pitfalls.

## Use of novel approaches in the diagnosis of schistosomiasis: Controversial results

When the first commercial version launched, POC-CCA acceptance was huge among field researchers. CCA can be detected by a labeled monoclonal antibody immobilized on the sample membrane. The solution then runs over the strip where the antigen–antibody complex attaches to another monoclonal antibody immobilized at the test line. The lateral flow assay is an easy to use test, which the first version worked by applying a single drop of urine with a buffer. Following 20 minutes of incubation, results were determined by visual reading (Schisto POC-CCA cassette based test; Rapid Medical Diagnostics, Pretoria, South Africa). Most data produced came from collaborative foreign research groups working in areas of moderate to high endemicity and populations infected with high parasite loads but also in low endemic areas as well as individuals with very light infection [5–10]. RTs and/or POCs may surpass conventional methods (such as the Kato-Katz test) as reliable diagnostic tools in moderate and high endemic areas. Yet variable results may also be found on a daily basis in these areas, and very low reactivity reactions in non–egg-excretors might misdiagnose active infection after praziquantel (PZQ) use [9]. Also, POC-CCA accuracy may be questionable in areas of *S*. *haematobium* mono infection or multiple *Schistosoma* infections [11–13]. But discussion becomes really intense when RTs are used in low parasite load and/or low endemic areas. Although several works consistently demonstrated that sensitivity, and also specificity, are compromised in low endemic areas, some new evidence claims that POC-CCA reliability and accuracy are high in areas of low prevalence [14–16]. Nonetheless, as in areas of high and moderate endemicity, residual reactivity (trace) was fairly demonstrated in individuals without active schistosomiasis both before and after (re)treatment in community settings with low parasite burden and/or low endemicity [16]. Usually, the bottleneck is the use of the low-sensitivity Kato-Katz test as a “gold standard.” Interpretation of trace as positive (presence of infection) or negative (absence of infection) creates lots of discussion about the validation of POC-CCA as a single test to replace Kato-Katz for the estimation of schistosomiasis prevalence in transmission areas. Particularly concerned with POC-CCA performance in low endemic areas, Clements and colleagues [17] evaluated trace results by applying Bayesian latent class analysis to improve the low sensitivity of the Kato-Katz and the so-called absence of a “gold standard.” In the same study, up-converting phosphor lateral flow circulating anodic antigen (UCP-LF CAA), a non-commercially available assay, was used to evaluate preselected trace-positive POC-CCA samples by assuming UCP-LF CAA was 100% specific. The results showed that POC-CCA outperformed Kato-Katz. However, considering trace as negative, *Schistosoma* active infection was missed in many cases. In contrast, trace as positive overestimated the prevalence. But by running UCP-LF CAA on selected trace-positive samples, the prevalence estimates from POC-CCA trace-positive samples should indeed represent the real prevalence in a lightly infected population. Recently, a new *S*. *mansoni* POC-CCA test became available, provided by only one manufacturer in the world. The new assay now uses only two drops of urine straight into a cassette instead of one drop of urine in addition to one drop of buffer as the recently discontinued previous version. The reading is done after 20 minutes like the older version. So far, no information was declared by the company regarding modifications on the test principle.

Despite the successful removal of traces, the elimination of potential false positivity resulted in no detection of true positives. Therefore, individuals with active schistosomiasis were misdiagnosed as negative by the new test version in low endemic areas. In the past, custom-tailored kits were tested, aiming to overcome the debatable accuracy and performance of POC-CCA in a low-prevalence population of school children [18]. Because only limited information was disclosed by the manufacturers, one assumes that changes on test sensitivity were also performed since the last commercially available assay.

One of the main reasons to adopt RTs as a diagnostic strategy is detection of active infection for surveillance purposes, including fast determination of prevalence in transmission areas and drug response. However, the POC-CCA for *S*. *mansoni* showed conflicting results depending on the area studied. In low endemic areas where low parasite loads or no egg-excretion predominates, POC-CCA was unsatisfactory as a solitary tool for diagnosis of active infections [15, 19]. Previous epidemiological studies in areas of high and moderate endemicity showed promising preliminary results during pretreatment with PZQ. However, persistence of very weak reactivity (trace) in the absence of egg excretion and/or other evidence of active infection could be seen in individuals treated with one to three rounds of PZQ [9]. Mwinzi and colleagues [20] showed that after the first round of PZQ use, 47% and 34% of POC-CCA reactive individuals ended up responding to second and third rounds of treatment, respectively, by becoming negative on POC-CCA. Nonetheless, more than 60% of retreated individuals still remained reactive. Other diagnostic tests had low agreement between pre- and post-therapy use, therefore the discussion remains about the usefulness of trace results. Recently, Coelho and colleagues [21] proposed optimization of POC-CCA in low–worm-burden samples by using lyophilization of urine as an antigen concentration method, showing that most of trace results turned negative and some negative samples became trace. In short, predefinition of trace as positive or negative may end up misleading test interpretation. In contrast, Prada and colleagues [22] addressed the true meaning of trace post-PZQ use and showed that POC-CCA seems to be a better predictor of post-treatment prevalence. Even trace results could be associated with true active infection. Following changes performed in RT manufacturing, as described above, very weak responses were swept out of the low endemicity areas, and no traces of “true” active infection could be detected. Now, the underscored results point to a possible underestimation of the real prevalence both pre- and post-treatments since traces disappeared. Without any consultation of the scientific community, the only available commercial test used so far in epidemiological studies changed its formulation and discontinued the previous test version (Schisto POC-CCA cassette based test; Rapid Medical Diagnostics, Pretoria, South Africa). No previous validation studies were performed in low endemic areas before commercial release of the test by the company. Also, the actual conflicting results do not permit decisions like the substitution of reference tests for POC-CCA.

## Conclusions and authors’ view

RTs showed high accuracy and performance in moderate and high transmission areas. The tests in platforms of point-of-care to diagnose *Schistosoma* infections are an appealing solution for mapping and surveillance of transmission areas because of their main characteristics: user-friendly and relatively low price. However, consistent evidence showed limited application of RTs in schistosomiasis diagnosis in low endemic areas. Mostly, in those settings, low parasite burden predominates, which results in a decrease of diagnostic test accuracy. Moreover, the presence of trace reactivity is a real pitfall. POC-CCA very low intensity reactions may hide low parasite–load-induced infections. Therefore, removal of trace might result in no detection of *Schistosoma* infection pre- and post-PZQ use and, as a result, improvement of the test must be encouraged. However, testing should precede the use by national programs. As it is, the diagnosis of *Schistosoma* infection pre- and post-therapy based solely on RTs and/or POC may be compromised in areas of controlled or eliminated schistosomiasis. Strategies based on a single commercial product without prevalidation in both low endemicity areas and in institutional settings can be worrisome. Blinded changes in RT formulation may end up increasing disaster situations and confusion. Commercially available diagnostic tools should be tested exhaustingly before release for general use in transmission and nonendemic areas worldwide.
